# Exploring the Association Between Time Dedicated to Lifelong Learning and Volunteering Among Older Adults: Socioeconomic Status as a Moderator

**DOI:** 10.1177/07334648251339513

**Published:** 2025-05-13

**Authors:** Juryung Kaitlyn Cho, Joonbeom Lee

**Affiliations:** 1 Department of Social Policy and Intervention, 6396 University of Oxford, Oxford, UK; 2 School of Public Service, 26729 Chung-Ang University, Seoul, South Korea

**Keywords:** active aging, lifelong learning, adult learning, education, social participation, civic engagement

## Abstract

This study explores the link between the time older adults spend on lifelong learning and volunteering, examining how this association varies by socioeconomic status (SES) in South Korea. Employing fixed-effects regression models, we analyze six waves (2008–2018) of panel data from the Korean Longitudinal Study of Aging (KLoSA). Findings reveal an inverse U-shaped relationship between the time spent in lifelong learning and volunteering, with the high SES group showing a sharper increase and quicker decline in the association compared to the low SES group. This study emphasizes the importance of educational programs in encouraging volunteering among older adults. Additionally, the findings suggest a necessity for support systems to be established to assist older adults, especially those with lower SES, who may encounter difficulties in volunteering despite their willingness to engage in such activities.

**What this paper adds**

• An inverse U-shaped relationship exists between the time spent on lifelong learning and volunteering among older adults in Korea, with SES playing a moderating role.
• The low SES group tends to alter their volunteering time at a relatively slower pace when the time spent on lifelong learning increases, compared to the high SES group.
• This study contributes to the active aging literature by showing that active aging is better understood through its interconnected components rather than in isolation.

**Application of study findings**

• Our results provide a nuanced understanding of the relationship between lifelong learning and volunteering, as well as the influence of SES on this relationship.
• Policymakers should consider the role of educational programs in community centers in encouraging volunteering among older adults, taking their socioeconomic diversity into account.
• The findings may help establish additional support systems to assist older adults, especially those who face challenges in participating and continuing to engage in social activities.

## Introduction

In light of the Active Aging framework, which promotes the idea of “optimizing opportunities for health, participation, and security to enhance the quality of life as people age” (World Health Organization, 2002, p. 12), many conceptual and empirical studies have been conducted on the topic of the benefits of various social participation activities (Narushima et al., 2018; Nyqvist et al., 2013). Lifelong learning and volunteering are particularly recognized as beneficial activities for social well-being (Merriam & Kee, 2014). Existing research highlights a positive link between lifelong learning, especially non-job-related, emotionally rewarding lifelong learning, and volunteering, as learning fosters self-confidence, empathy, and social networks, which promote civic participation (Sung et al., 2023; Wilson, 2000).

However, coherent evidence is scarce regarding the degree to which adult learning affects civic engagement (Rüber et al., 2018). Furthermore, the question remains as to whether the positive relationship between the time spent in education and volunteering is consistent. Given that time is a limited resource, the question is not simply one of whether someone volunteers, but also how much time they commit. It is particularly important when older adults are involved in both lifelong learning and volunteering, as the time that older adults can dedicate to these activities not only contributes to their own cognitive and emotional well-being but also to the success and sustainability of participating in such activities.

In this study, we thus examine the relationship between the time older adults spend on volunteering and lifelong learning. Volunteering has been suggested as an essential measure of civic engagement (Stadelmann-Steffen & Freitag, 2011) because it contributes to the achievement of community sustainability (Portney, 2005). Lifelong learning can be defined as the “continuing development of knowledge and skills that people experience after formal education and throughout their lives” (London, 2011, p. 4). Lifelong learning is typically categorized into formal, non-formal, and informal types. Formal learning occurs in educational institutions with a set curriculum and leads to qualifications. Non-formal learning takes place outside formal settings, is often organized but voluntary, and lacks certification. Finally, informal learning is unstructured, spontaneous, and embedded in daily life experiences without institutional involvement (Nygren et al., 2019). This study focuses on non-occupational, non-formal lifelong learning when examining its association with volunteering, as it is considered to be more emotionally rewarding compared to job-related lifelong learning, which tends to be instrumental and goal-oriented (Sung et al., 2023).

This paper is guided by the Socioemotional Selectivity Theory (Carstensen et al., 1999), which suggests that people prioritize emotionally meaningful activities because of limited time. It also draws on the empowerment theory (Zimmerman & Rappaport, 1988) and the transformative learning theory (Mezirow, 1991), which link learning process to public engagement. With the above in mind, we posit that the emotional fulfillment and empowerment gained through lifelong learning foster a positive association with volunteering. We further posit, using the allocation of time theory (Becker, 1965), that while participation in educational programs can initially encourage volunteering, time and resource constraints may limit this positive association, eventually leading to a negative association as time commitments increase. We also hypothesize that this association varies by the socioeconomic status (hereafter: SES) of older people. Scholars argue that older adults, as a heterogeneous group, have different motivations for participating in educational programs and face distinct challenges in volunteering, largely shaped by their SES (Choi & Chou, 2010; O'Dea et al., 2023). Resource theory suggests that access to resources such as time, money, and civic skills significantly influences participation in lifelong learning and volunteering, highlighting disparities in civic engagement (Lancee & Van de Werfhorst, 2012). Therefore, we predict that increased time spent on lifelong learning will result in fewer positive changes in volunteering time for lower SES older adults due to their limited resources compared to their higher SES peers.

We examine these hypotheses using six waves of longitudinal data from a nationally representative sample of older adults in South Korea (hereafter: Korea). The Active Aging Index (AAI) measures older adults’ engagement across four domains: employment, social participation, independent living, and capacity for active aging (Zaidi et al., 2017). In Korea, AAI results indicate strong performance in employment but weaker outcomes in social participation and independent living compared to the EU and China (Um et al., 2019). Korea is the fastest-aging country in the world (Kim & Kim, 2020). However, social participation, such as lifelong learning and volunteering, has not traditionally been a familiar concept to many older adults in Korea, which may have much to do with historically limited welfare provisions. Given the relatively late introduction of the National Pension Scheme in 1998—Korea’s principal social insurance-based public pension program—only 32.3% of older adults were receiving benefits by 2013 (Ku & Kim, 2020). Although this figure increased to 51.2% by 2023 (National Pension Service, 2023), many older people remain without public pension support. Older individuals in Korea generally have extremely low levels of income and education, which may hinder their ability to engage in social participation. Korea has had the highest old-age poverty rate for over 15 years, reaching 43.4% in 2018 compared to the Organisation for Economic Co-operation and Development (OECD) average of 13.1% in the same year (OECD, 2021). Educational attainment also remains low, with approximately 43% of older adults having completed only elementary education or less (OECD, 2023). These constraints—financial hardship and low education levels—may severely limit their capacity to participate in social activities. This is reflected in high rates of old-age poverty, significant income inequality, and many older adults remaining in the labor market due to insufficient pensions (Chae & Heshmati, 2024). In Korea, older adults aged 60 and above make up only 9% of all volunteers (Lee & Jean Yeung, 2019). This represents a relatively low proportion compared to countries with stronger welfare provisions and lower poverty rates among older adults, where volunteering is more common in later life (Um et al., 2019).

Despite the crucial role of resources in older adults’ lifelong learning and volunteering, there is limited research on whether and how participation in educational programs correlates with volunteer activities in contexts where such resources are scarce among older individuals. To address this gap, it is crucial to examine how time allocation for these activities differs among older adults of varying SES, particularly in contexts such as Korea where resources are limited among older people. While direct interventions can encourage volunteering, this paper examines how non-vocational lifelong learning may foster positive engagement, offering a potentially more sustainable and empowering pathway through personal growth and emotional fulfillment. This study offers long-term and repeatable implications for countries facing similar challenges across the world, such as rapid population aging, high poverty, and low education levels among older adults.

## Theories and Hypotheses

### Socioemotional Selectivity Theory and the Empowering Nexus Between Lifelong Learning and Volunteering

The Socioemotional Selectivity Theory (SST) (Carstensen et al., 1999) offers valuable insights into the positive relationship between lifelong learning and volunteering. The SST suggests that as individuals grow more conscious of the limited time left in their later years, they tend to place greater importance on activities that hold emotional significance (Carstensen, 1992). Generativity, as an emotionally significant pursuit, reflects the desire to leave meaningful legacies and contribute to the well-being of future generations (Erikson, 1963). A key driver of generativity is the sense of inclusion and belonging (Villar et al., 2023). In line with the SST (Carstensen et al., 1999), participation in emotionally meaningful lifelong learning programs can strengthen a sense of inclusion and belonging, which may naturally encourage generativity and engagement in volunteering. Older lifelong learners are also known to exhibit generative motivations that align with volunteering (Yamashita et al., 2017).

In addition to the SST, the empowerment theory (Rappaport, 1987) provides valuable perspectives into the positive relationship between lifelong learning and volunteering. Previous studies demonstrated that empowerment is a critical factor in increasing civic engagement (Chan & Mak, 2020; Zimmerman & Rappaport, 1988). Empowerment refers to the process of linking a sense of belongingness and agency with the desire to engage in the public sphere (Chan & Mak, 2020). Research also suggests that the impact of empowered older individuals on their civic engagement stems from self-affirmation through successful identification of their social role and fulfilling their desire for belongingness to society (Lorenz, 2013). Providing emotionally meaningful educational programs for older adults aligns with the empowerment theory, which helps the cultivation of strengths and capabilities within individuals (Cox, 1989; Lloyd, 1991; Perkins & Zimmerman, 1995). Transformative learning theory (Mezirow, 1991) explains the transformative process adults undergo during learning, enabling them to reflect on their knowledge and perspectives while reconsidering their values. It emphasizes that adult learning promotes self-development, encouraging the exploration of new roles and actions (Mezirow & Taylor, 2009). According to the need hierarchy (Maslow, 1987), every individual has an innate desire and need for self-realization, which can be satisfied through personal development. In particular, when considering older people, creating meanings about the self in the realm of social status could be a source of satisfaction and meaning in their lives, particularly as they navigate changes in social roles and cope with various losses, including the loss of roles, in later life (Minkler, 1981).

Alongside the theoretical frameworks suggested above, several empirical studies have substantiated the positive link between lifelong learning and volunteering. Empirical findings by Tett and Maclachlan (2007) demonstrate that people who took either numeracy or literacy courses reported higher levels of civic engagement as a result of their improved sense of self, with the additional positive outcome of an expanded network of friends and acquaintances. Preston (2004) highlights enhanced confidence as a potential outcome of learning, which may mediate the relationship between education and civic engagement. Furthermore, it has been suggested that people empowered through personal development are likely to go on to pursue civic engagement and consider being a volunteer as one’s primary role after retirement (Kaskie et al., 2008).

Based on the studies suggested here, we expect that lifelong learning may boost the belongingness and personal growth of older adults and increase generativity, which may then be associated positively with the civic engagement of older people. In addition, using empowerment theory, we posit that older adults who are empowered through the process of personal development would be more motivated to participate in civic engagement as learning new experiences and skills can help individuals exercise knowledge and skills. Viewed through the lenses of the SST and empowerment theory, we can suggest with confidence a potential positive association between lifelong learning and volunteering as indicated in many literatures.

### Balancing Lifelong Learning and Volunteering: Exploring the Time Allocation

Using the SST and empowerment theory, we suggested above that there may be a positive relationship between personal development and civic engagement. Our study centers on the relationship between participation in lifelong learning and volunteering, specifically examining the hours dedicated to these two activities. Given that time is a finite resource, the allocation of time theory (Becker, 1965), which examines work and leisure, can be adapted to lifelong learning and volunteering by recognizing that individuals internally weigh the trade-offs between time spent on different activities, factoring in opportunity costs. This suggests that we cannot anticipate a consistently positive correlation between the time invested in both activities. Also, it has been suggested that time constraints and a greater priority associated with another productive activity or commitment may cause older persons to disengage from or discontinue their participation in a volunteer program (Tang et al., 2010). We expect that if older adults are offered the opportunity to develop their skills further by participating in educational programs, they will volunteer more. However, due to time and resource constraints and the difficulties older adults face in balancing volunteering with other commitments, the positive association may be limited to a certain range, beyond which the association is expected to become negative. We thus hypothesize that *there will be a positive association between time allocated to lifelong learning and volunteering. Note, however, that this positive relationship is expected to be constrained to a limited range of time, after which the association is likely to become negative (H1)*.

### Socioeconomic Status (SES) and the Varying Levels of Resources Available to Older Adults

Given our examination of the Korean context, characterized by high poverty rates and inequality among older people, we must delve into different literature sources to understand the potential for varying levels of empowerment among older individuals below. Studies suggesting that volunteering motivation and barriers change with SES (Golinowska et al., 2016) point to a possible variation in the relationship according to SES. Scholars suggest that older adults are a heterogeneous population who share different motivations for participating in educational programs and barriers to continued civic engagement, which may depend on one’s SES (Choi & Chou, 2010; Gray et al., 2016; O'Dea et al., 2023).

Resource theory suggests that older adults with strong human capital, social networks, and positive psychological traits are more likely to participate in lifelong learning and volunteering (Lancee & Van de Werfhorst, 2012; Sung et al., 2023). It has been suggested that a higher SES is associated with greater life satisfaction and a stronger sense of belonging to society, while a lower SES is linked to lower self-confidence and increased depressive symptoms (Miech & Shanahan, 2000). Lifelong learning, as discussed, is expected to empower older adults by enhancing their skills and confidence, thereby increasing their motivation for social contribution. However, we expect that empowerment is likely to be more pronounced among those with higher SES, as their elevated life satisfaction and sense of hierarchy can create a synergy between personal development and volunteering. In contrast, for older adults with lower SES, limited resources and less favorable psychological conditions may hinder the development of such a synergistic effect through lifelong learning alone.

Tang and her colleagues (2010) find that older individuals with lower SES often discontinue volunteering due to challenges such as inadequate transportation, poor health, and conflicting responsibilities, including caregiving and employment obligations for their families or dependents. When voluntary organizations or programs fail to provide sufficient support, even low SES older adults already engaged in volunteering may struggle to sustain their participation over time. Time is a critical resource, and lower SES groups face greater pressures from time constraints, particularly as they invest more time in personal development (Dury et al., 2015). Economic activities, such as paid work, can act as spatiotemporal barriers that reduce the time available for volunteering (Lockstone-Binney et al., 2010). For lower SES older adults, time spent on earning a livelihood often takes priority, leaving little room for civic engagement (Principi et al., 2012). Building on these insights, we anticipate in Hypothesis 1 that older adults’ civic engagement time will initially increase but eventually decline. We further hypothesize that *due to differing motivations and resources across SES levels, increases in personal development time will have a less positive association with time spent on civic engagement for lower SES older adults compared to their higher SES counterparts (H2)*.

## Methods

### Data and Sample

The main dataset examined for our study comes from the Korean Longitudinal Study of Aging (KLoSA). The target population of the KLoSA is people who are aged 45 years or older in Korea, and KLoSA has a baseline sample of 10,248 respondents. The KLoSA is approved by the Korea National Statistical Office (approval number: 33602) and is publicly available as provided by the official governmental agency, the Korea Employment Information Service (KEIS).

### Variables

#### Dependent Variable: Volunteering

Volunteering is measured as the average number of hours per month spent on volunteering activities in the past year. In the questionnaire, volunteering activity refers to the work of participating in government agencies, religious organizations, civic societies, and clubs, doing meaningful work or helping people in need without compensation.

#### Independent Variable: Lifelong Learning

Lifelong learning is measured as average number of hours per month spent on educational programs provided by local centers (i.e., agencies acting on behalf of the central or local government) such as community service centers, district offices, county offices, city halls, and welfare centers. In terms of educational programs offered, these include language courses such as Korean, Chinese characters, and other foreign languages, as well as digital skills education.

#### Moderating Variable: SES

The KLoSA survey measures self-perceived SES using a 6-point scale, ranging from 1 to 6. According to the questionnaire and our original dataset, a higher value indicates a lower status. To enhance readability, the responses were reversely coded. In other words, the minimum value 1 indicates the lowest status and the maximum value 6 corresponds to the highest status. For a clear comparison, we combined the first three categories to form the “low SES” group and the last three categories as the “high SES” group, creating two distinct categories.

### Covariates

Drawing on insights from previous studies (e.g., Grizzle, 2015; Komp et al., 2012), we include several covariates in the model to better capture the factors influencing our outcomes and control for potential confounding variables: gender, age, and marital status, as well as subjective health status, measured using a five-point Likert scale. These factors are known to influence social participation and engagement. Furthermore, we include economic variables such as total income and personal economic situation, categorized as employment, unemployment, or non-economic activity, as these variables reflect access to resources and opportunities that may shape engagement behavior (Principi et al., 2016). A detailed explanation and descriptive statistics of the variables used in our study can be found in Table 1.

**Table 1.** Descriptive Statistics, KLoSA.

Variable	Variable Type	Obs	Mean	Std. Dev.	Min	Max
Volunteering	Continuous	40,181	0.343	2.997	0	80
Lifelong learning	Continuous	40,181	0.11	1.647	0	80
SES	Continuous	40,172	2.677	1.092	1	6
Gender	Dichotomous	40,181	0.44			
Age	Continuous	40,181	64.686	8.481	50	80
Marital status	Dichotomous	39,272	0.003			
Subjective health status	Continuous	40,181	3.657	0.893	1	5
Economic situation	Categorical	40,180	2.108	0.99		
Total income (in log)	Continuous	39,739	5.92	2.259	−.693	13.127

*Note.* Our panel dataset includes a baseline of 9124 individuals, comprising a total of 38,834 observations. The SES variable is classified as 1 (lower low, 16.99%), 2 (upper low, 25.16%), 3 (lower medium, 35.11%), 4 (upper medium, 19.26%), 5 (lower high, 2.8%), and 6 (upper high, 0.67%). The gender variable is coded as 0 (woman, 56.03%) and 1 (man, 43.97%). Marital status is coded as 0 (same as the previous survey, 99.67%) and 1 (different from the previous survey, 0.34%). Subjective health status is categorized as 1 (employed, 44.24%), 2 (unemployed, 0.76%), and 3 (economically inactive, 55%).

### Strategy of Analysis

We employ fixed-effects regression models (Brüderl & Ludwig, 2015) as our primary analytical approach (Table 2) and use random-effects models for robustness checks (Appendix 1). The panel fixed-effects model has the advantage of controlling for unobserved, time-invariant individual characteristics. In Models 1 and 2, we test a linear relationship between volunteering hours and lifelong learning hours, and the relationship between volunteering hours and SES, respectively. In Models 3 and 4, we test the quadratic relationship between lifelong learning hours and volunteering hours. In Model 4, we specifically examine the interactive relationship between lifelong learning hours and SES.

**Table 2.** Fixed-Effects Regression of Volunteering Hours on Lifelong Learning Hours.

Variables	(1)	(2)	(3)	(4)
	Model 1	Model 2	Model 3	Model 4
Lifelong learning	0.0334***	0.0334***	0.0997***	−0.0791
	(0.0119)	(0.0119)	(0.0211)	(0.0700)
Lifelong learning (squared term)			−0.000580***	0.000313
			(0.000153)	(0.000812)
Socioeconomic status (SES)		0.0743**	0.0747**	0.0698*
		(0.0380)	(0.0380)	(0.0380)
Lifelong learning * SES				0.0576***
				(0.0213)
Lifelong learning (squared term) * SES				−0.000289***
				(0.000243)
Gender				
Age	−0.0534***	−0.0526***	−0.0525***	−0.0525***
	(0.00915)	(0.00916)	(0.00915)	(0.00915)
Marital status	−0.0307	−0.0293	−0.0192	−0.0248
	(0.474)	(0.474)	(0.474)	(0.474)
Subjective health status	−0.104**	−0.0969**	−0.0956**	−0.0957**
	(0.0415)	(0.0417)	(0.0417)	(0.0417)
Economic situation (ref. = employed)				
Unemployed	0.477	0.492	0.487	0.482
	(0.320)	(0.320)	(0.320)	(0.320)
Economically inactive	0.292***	0.294***	0.292***	0.294***
	(0.0973)	(0.0973)	(0.0972)	(0.0972)
Total income (in log)	0.0271	0.0268	0.0272	0.0270
	(0.0167)	(0.0167)	(0.0167)	(0.0167)
Constant	4.031***	3.760***	3.736***	3.754***
	(0.586)	(0.602)	(0.602)	(0.602)
Observations	38,864	38,856	38,856	38,856
R-squared	0.002	0.003	0.003	0.003
F-statistics	8.04***	8.07***	8.53***	8.48***
Number of individuals	9124	9124	9124	9124

Robust standard errors in parentheses.

***p < .01, **p < .05, *p < .1.

## Results

Table 2 presents estimates of the association between hours spent on lifelong learning and volunteering. All models control for gender, age, marital status, subjective health, economic situation, and total income. Model 1 includes the linear term of lifelong learning hours along with all covariates, showing a positive linear relationship between lifelong learning and volunteering (b = 0.0334, *p* < .01). In Model 2, SES is included and shows a significant positive association with the time spent on volunteering (b = 0.0743, *p* < .05), and the positive association with lifelong learning hours remains unchanged (b = 0.0334, *p* < .01).

In Models 3 and 4, we introduce the squared term of lifelong learning hours. With respect to the estimates of lifelong learning hours and SES, we find a significant quadratic relationship between time spent on lifelong learning and volunteering, regardless of whether SES is included as an interactive term.

In Model 3, the estimated value of the squared term of lifelong learning is negative and statistically significant (b = −0.000,580, *p* < .01), indicating an inverted U-shaped relationship between lifelong learning and volunteering. This analysis suggests that a one-unit increase in time invested in lifelong learning leads to an increase in volunteering hours; however, beyond a certain inflection point, volunteering hours begin to decline.

Finally, in Model 4, we find that the SES moderates the inverted U-shaped relationship between time spent on lifelong learning and volunteering. The coefficient of the interaction term between squared lifelong learning hours and SES is negative and significant (b = −0.000,289, *p* < .01). The results from the panel regression with a random-effects model, presented in Appendix 1 as a robustness check, also show that the same coefficient is negative and significant (b = −0.000,289, *p* < .01).

**Figure 1.**
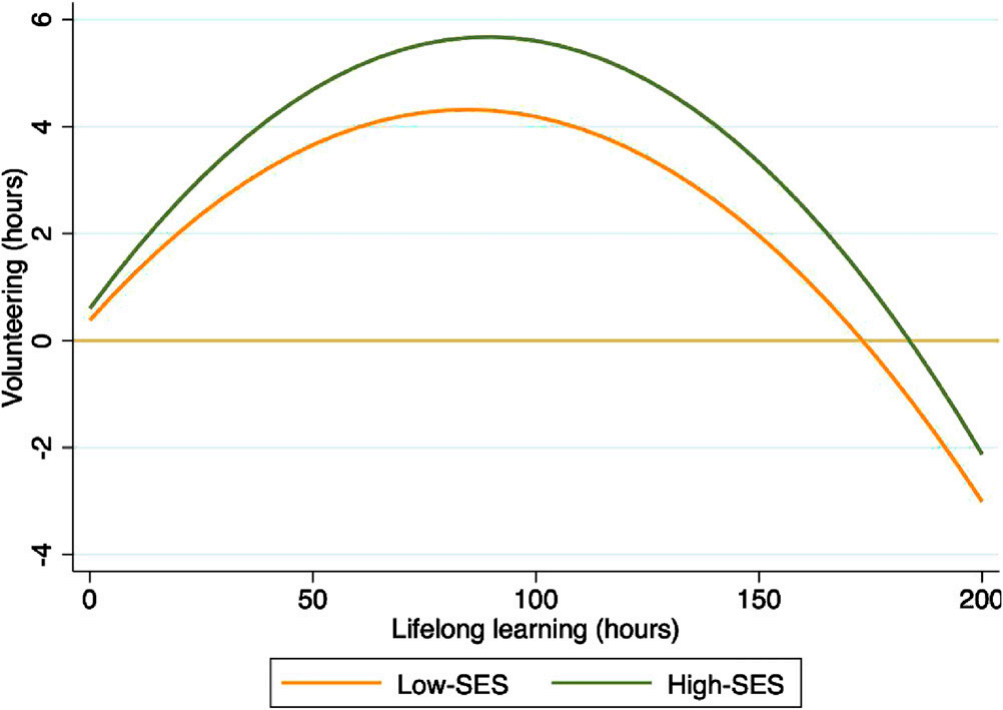
Inverted U-shaped relationship between lifelong learning hours and volunteering hours, by socioeconomic status.

Figure 1 shows that the high SES group is much more responsive to changes in volunteering hours as the time invested in lifelong learning increases, compared to the low SES group. The curves show that the high SES group reaches a higher peak in volunteering hours and exhibits greater susceptibility to changing their time spent on volunteering compared to the low SES group. The low SES group shows a tendency to alter their volunteering time at a relatively slower rate, when the time spent on lifelong learning increases, compared to the high SES group.

## Discussion and Conclusion

Using the national longitudinal data, we observed a reverse U-shaped relationship between time spent on lifelong learning and volunteering, which differs based on the SES levels of older adults. For H1, we proposed that the linear relationship might not remain stable and that there could be a point in which the time spent in lifelong learning and volunteering decreases. One could reasonably assume that this is noticeable as individuals are expected to balance their limited time and prioritize between competing commitments. Our findings support this hypothesis. The positive association observed in the initial range adds to and enhances existing research on the various connections between lifelong learning and volunteering. Based on the SST, empowerment theory, and transformative learning theory, it is reasonable to suggest that the process of empowerment through learning, coupled with generativity in later life, facilitates participation in both activities, although these mechanisms could not be directly tested in our study. Future studies should aim to empirically explore the pathways through which lifelong learning impacts volunteering. Our findings also support H2, showing that the reverse U-shaped relationship between time spent on lifelong learning and volunteering is more pronounced among higher SES groups compared to lower SES groups. This aligns with resource theory, as while greater generativity may occur in later life, limited resources can weaken the link between lifelong learning and volunteering. High SES individuals are likely to possess more resources and flexibility, enabling them to volunteer more easily when engaged in lifelong learning. In contrast, individuals with low SES, limited by resource constraints, may struggle to easily engage in volunteering while participating in lifelong learning.

To the authors’ knowledge, this paper is the first of its kind which attempts to analyze the association between time spent in lifelong learning and volunteering and the moderating role of SES in that relationship. This study builds on existing research demonstrating a positive association between informal lifelong learning and volunteering (Sung et al., 2023; Yamashita et al., 2017) while also identifying trade-offs between the two activities. This study therefore highlights the need to consider structural constraints and variations among older adults, such as time limitations and SES disparities, all of which are bound to shape engagement patterns. In doing so, it contributes to the active aging literature by demonstrating that active aging is best understood through the interplay of its components rather than viewing each—such as volunteering or lifelong learning—as an independent factor (Boudiny, 2013; Santini et al., 2020; Timonen, 2017). Furthermore, this study offers a more inclusive framework upon which one could establish a deeper understanding of active aging by shifting the focus from whether older adults participate in different activities to how much time they can dedicate to different activities with a focus on SES disparities. This study bridges empowerment theory and the active aging literature by examining how socioeconomic disparities relate to the distribution of time across different forms of participation, offering a careful examination of how the empowerment process may vary depending on socioeconomic resources and constraints. The consideration of all these factors may be essential for ensuring that older adults have the time and capacity to engage—particularly in low-resource settings—rather than assuming that volunteering in later life depends primarily on personal motivation.

Our findings provide insights for active aging policies, emphasizing the need for targeted, flexible approaches for social activities that address socioeconomic disparities among older people. Since SES plays a crucial role in shaping the connection between time invested in lifelong learning and volunteering, customized approaches are likely to yield the most effective and affective outcomes. For higher SES individuals, policies could focus on flexible scheduling and institutional support, such as hybrid learning options to enable them to sustain volunteering while engaging in lifelong learning. Conversely, for those with lower SES, blending volunteer opportunities into lifelong learning curricula—such as through service-learning models—could present the most practical way to encourage involvement without adding extra time burdens (Yamashita et al., 2017). Additionally, recommending volunteer activities that match the content learned in lifelong education or those that align with personal interests and abilities can help lower the participation barrier. Through individual counseling, lifelong learning facilitators can pinpoint—such as physical limitations, anxiety, or insufficient information—and then go on to provide customized solutions. Additionally, removing practical barriers such as transportation costs, health limitations, and caregiving responsibilities could further enable participation, particularly for those with fewer resources. However, the key is that older adults should not be compelled nor expected to participate in both activities; support should be provided based on their independent and self-motivated choice to engage (Stephens et al., 2015).

In line with numerous empirical studies, this article has limitations. The most significant of which is the inability to examine the mechanisms underlying the relationship between personal development and civic engagement. Additionally, we cannot entirely rule out the influence of unidentified confounding factors. While we expect generativity and empowerment to contribute to positive association in the initial range of the inverse U-shaped relationship, these factors were not directly measured. However, what this study attempts to do is to mitigate some of those limitations by utilizing six waves of panel data, which is intended to provide more robust estimates and effectively control for unobserved confounders that cross-sectional or time-series data cannot capture. Further, we were unable to discern the purpose behind participation in lifelong learning and volunteering due to a lack of data. Future research could address this gap by exploring individuals’ motivations for engaging in or withdrawing from these activities. Lastly, as this study utilizes data from the Korean Longitudinal Study of Aging (KLoSA), the findings may reflect characteristics specific to the Korean context, in which high poverty rates, income inequality, and financial pressures often limit older adults’ engagement in lifelong learning and volunteering. Nevertheless, these insights must at least in part be relevant for countries facing similar socioeconomic challenges, suggesting the need for targeted support that accounts for SES differences to promote meaningful participation. Future research could investigate whether the observed inverse U-shaped relationship between the time spent on personal development and civic engagement holds across diverse sociocultural contexts and further explore the mechanisms underlying this association. Despite these limitations, this study highlights the interplay among older adults’ participation in lifelong learning, volunteering, and SES levels, providing valuable policy insights to promote a more holistic and inclusive approach to active aging.

## Supplemental Material

Supplemental Material - Exploring the Association Between Time Dedicated to Lifelong Learning and Volunteering Among Older Adults: Socioeconomic Status as a Moderator Supplemental Material for Exploring the Association Between Time Dedicated to Lifelong Learning and Volunteering Among Older Adults: Socioeconomic Status as a Moderator by Juryung Kaitlyn Cho and Joonbeom Lee in Journal of Applied Gerontology.

## Data Availability

This article does not report data and therefore the pre-registration and data availability requirements are not applicable.*
