# Supplementary material for: Exploring the Association Between Time Dedicated to Lifelong Learning and Volunteering Among Older Adults: Socioeconomic Status as a Moderator
Source: J Appl Gerontol. 2025 May 13;45(2):279–88. doi: 10.1177/07334648251339513 (PMC12759100; doi:10.1177/07334648251339513)
Supplement: Supplemental Material - Exploring the Association Between Time Dedicated to Lifelong Learning and Volunteering Among Older Adults: Socioeconomic Status as a Moderator [file sj-pdf-1-jag-10.1177_07334648251339513.pdf]

Appendix 1. Random Effects Regression of Volunteering Hours on Lifelong Learning Hours

VARIABLES	(1)<br>Model 1	(2)<br>Model 2	(3)<br>Model 3	(4)<br>Model 4
Lifelong learning	0.0604***	0.0593***	0.178***	-0.330***
	(0.00904)	(0.00904)	(0.0182)	(0.0675)
Lifelong learning (squared term)			-0.000315***	0.000581***
			(0.000422)	(0.000210)
Social Economic Status (SES)		0.135***	0.135***	0.127***
		(0.0159)	(0.0159)	(0.0157)
Lifelong learning * SES				0.166***
				(0.0204)
Lifelong learning (squared term) * SES				-0.000289***
				(0.000623)
Gender	-0.193***	-0.203***	-0.201***	-0.203***
	(0.0411)	(0.0410)	(0.0407)	(0.0395)
Age	-0.0106***	-0.00846***	-0.00848***	-0.00837***
	(0.00247)	(0.00247)	(0.00246)	(0.00240)
Marital Status	0.121	0.136	0.144	0.135
	(0.253)	(0.253)	(0.253)	(0.253)
Subjective health status	-0.105***	-0.0669***	-0.0668***	-0.0701***
	(0.0193)	(0.0198)	(0.0197)	(0.0196)
Economic situation (ref.= Employed)				
Unemployed	0.403**	0.452***	0.457***	0.447***
	(0.169)	(0.169)	(0.169)	(0.169)
Economically inactive	0.245***	0.235***	0.232***	0.236***
	(0.0403)	(0.0403)	(0.0401)	(0.0396)
Total income (in log)	0.00872	0.00533	0.00517	0.00494
	(0.00781)	(0.00781)	(0.00780)	(0.00776)
Constant	1.354***	0.759***	0.758***	0.782***
	(0.151)	(0.166)	(0.165)	(0.162)
Observations	38,834	38,826	38,826	38,826
Wald chi2	207.68***	280.84***	340.02***	453.89***
Number of individuals	9,124	9,124	9,124	9,124

Robust standard errors in parentheses
*** p<0.01, ** p<0.05, * p<0.1
